# A Case Report on Isolation of Linezolid- and Vancomycin-resistant Enterococcus faecium Species from Cerebrospinal Fluid of a Patient Suffering from Ventriculoperitoneal Shunt-associated Meningitis

**DOI:** 10.7759/cureus.5631

**Published:** 2019-09-12

**Authors:** Seema Irfan, Salima Qamar

**Affiliations:** 1 Microbiology, Aga Khan University Hospital, Karachi, PAK

**Keywords:** linezolid, vancomycin, meningitis, enterococcus faecium species, drug resistance, pakistan

## Abstract

Vancomycin-resistant enterococci (VRE) are one of the most common nosocomial infections. Linezolid has been used to treat such infections extensively. Over time there have been reports where linezolid resistance in enterococci has been documented. This is the first report from Pakistan where linezolid- and vancomycin-resistant Enterococcus faecium was isolated from cerebrospinal fluid (CSF) sample from infected ventriculoperitoneal (VP) shunt.

## Introduction

Enterococci are natural commensals of the human gastrointestinal tract. The most common species associated with nosocomial infection are Enterococcus faecalis and Enterococcus faecium. These enterococci sometime acquire resistance to vancomycin, hence termed as vancomycin-resistant enterococcus (VRE). VRE is a difficult to treat nosocomial organism that is a threat to infection control as well. This organism can spread from one person to another via contact, especially in healthcare setting where it can cause significant morbidity in immune-compromised population. It is implicated in a variety of infections like urinary, abdominal and line-related infections. It is usually treated with agents like daptomycin, ampicillin, chloramphenicol, quinupristin/dalfopristin, nitrofurantoin, fosfomycin and linezolid [1]. Since daptomycin and quinupristin-dalfopristin are not available in Pakistan, clinical microbiology laboratory of Aga Khan University Hospital (AKUH) does not perform susceptibility against these drugs. Out of these, linezolid is the most commonly used antibiotic to treat VRE infections in our settings. This antibiotic preference is due to its good tissue penetration capability plus cost effectiveness and availability. Here we report a case of a young male with ventriculoperitoneal shunt infection caused by linezolid-and vancomycin-resistant Enterococcus faecium species (LRVRE).

## Case presentation

On 26th June 2016, a cerebrospinal fluid (CSF) sample from a 17-year-old male was received in microbiology section at clinical laboratories, Aga Khan University Hospital, Karachi (AKUH). This laboratory receives clinical specimens from both AKUH inpatients as well as from all over the country. This sample was sent from a private hospital of Lahore city. It was requested for CSF detailed examination, gram stain, routine bacterial culture and sensitivity. Physician was contacted on telephone and patient’s clinical history was taken, plus advice on management was given as a part of routine clinical microbiology reporting. History revealed that boy had a road traffic accident two months back, got multiple injuries involving head, chest and limbs. He was treated for various injuries involving chest and limbs and was started on meropenem and vancomycin empirically due to his critical condition along with having all the initial microbiological cultures negative. After nearly three weeks, he developed hydrocephalus for which ventriculoperitoneal (VP) shunt was placed. Nearly a week following the shunt procedure his GCS dropped and his antibiotics were changed, meropenem was continued, however, vancomycin was replaced with linezolid, amikacin and metronidazole.

His CSF DR showed glucose 54.6 mg/dl, protein 120 mg/dl, RBCs 150/cu mm, TLC 143/cu mm with poly 90% polymorphs. His CSF gram stain showed few pus cells and no microorganism. Routine bacterial culture of CSF was inoculated on chocolate agar and sheep blood agar (SBA) that were placed in 5% CO2 at 35-37C for 24-48 hours. MacConkey’s agar was also set and incubated aerobically at 35-37C for 24-48 hours. In addition to solid culture media a 5-ml tube of brain heart infusion (BHI) broth was also inoculated with few drops of CSF and kept aerobically at 35-37C for 24-48 hours. After 24 hours his culture grew alpha-hemolytic streptococci on primary plates. It was identified as Enterococcus faecium using API streps (bioMérieux, France) as well as Vitek 2 (bioMérieux, France). Susceptibility performed on Muller Hinton agar showed it to be resistant to ampicillin (10 µg), chloramphenicol (30 µg), vancomycin (30 µg). It was further tested for linezolid (30 µg), fosfomycin (200 µg) and tigecycline (15 µg) as second line agents which showed it to be resistant to linezolid and fosfomycin and sensitive to tigecycline. Finally MIC break points were evaluated for all above antibiotics including vancomycin >=32 µg/ml, teicoplanin >=32 µg/ml and linezolid >8 µg/ml, using Vitek 2 system (bioMérieux, France) while this E. faecium strain was found susceptible to tigecycline with MIC of 0.12 μg/ml. Broth micro dilution was not performed for any antibiotic. Telephonic communication between the physician and microbiologist was maintained throughout the sample processing till results were finalized. Based on available sensitivities, treatment with tigecycline was suggested along with contact precautions after discussion with an infectious disease physician along with removal or change of infected shunt. Suggestions regarding LRVRE source evaluation were communicated to primary physician, however, due to lack of resources, this could not be done. Unfortunately, communication with the primary physician was also lost after few days, therefore, final outcome of the patient remained unclear.

## Discussion

Linezolid resistance amongst VRE has been reported previously. In 2001, Gonzales et al. reported cases where linezolid-resistant VRE were isolated from different sites in five patients in three hospitals over the course of three months [1]. Four out of these five patients were transplant recipients who were already on linezolid. After linezolid resistant VRE were identified they were switched to quinupristin-dalfopristin. In 2002, first case of linezolid- and vancomycin-resistant enterococcus was reported from the UK [2]. Point mutation in 23S rRNA was identified as the cause of emerging linezolid resistance. Prystowsky in 2001 performed DNA sequencing and identified linezolid resistance in three E. faecalis isolates to be associated with a guanine to uracil transversion at bp 2576 and one E. faecium isolate with MIC 16 μg/ml containing a guanine to adenine transition at bp 2505 [3]. Since then similar strains emerged from different parts of the world like Germany, United states and Brazil [4-6]. Although this patient had been given linezolid during his hospital stay, the mode of acquisition of acquired resistance gene was not evaluated. We did not perform sequencing to determine the exact mutation associated with resistance to linezolid in this strain due to financial restrains and unavailability of the needed methodology in our laboratory. Therefore, we can only hypothesize that this strain had developed resistance mutation during linezolid therapy.

To the best of our knowledge, there has been no report about linezolid resistance in VRE from our country. We isolated linezolid resistant VRE in a CSF sample with a significant CSF DR findings and foreign device in place. Since daptomycin and quinupristin-dalfopristin are not available in Pakistan and we did not have any methods of determining its susceptibility, it was not evaluated. Tigecycline was the only available antibiotic against which this E. faecium was susceptible, however, this drug has poor penetration in the CSF. Keeping in consideration of this limitation, an expert opinion was sought from AKUH infectious disease consultants. Since there were no antimicrobial options left to treat current strain of E. faecium, tigecycline was suggested to be added to the patient’s therapy. Literature search also revealed recently published report showing successful treatment with intravenous and intraventricular tigecycline of a case of ventriculoperitoneal shunt-related infection caused by daptomycin resistant E. faecium [7]. Since CLSI does not provide tigecycline cutoffs for enterococcus species, EUCAST cutoffs were adopted which showed our strain with MIC 0.12 μg/ml to be sensitive. Our strain was also tested for chloramphenicol, teicoplanin and fosfomycin, however, it was found to be resistant. One of limitations of our study is failure to obtain final outcome of patient. As a patient was admitted in one of the private tertiary health care setting of Lahore city and clinical microbiology laboratory of AKUH is located in Karachi, therefore it was difficult for us to maintain continuous progress of the patient via telephonic contact between clinical microbiologist and physician. Hence, ultimate outcome of the patient is not known as communication was lost after few days, although we made constant efforts to reach patients’ team. Under usual circumstances isolation of vancomycin-resistant enterococci in a healthcare setting dictates strict use of contact precautions. However, isolation of linezolid-resistant vancomycin-resistant enterococci demands not just contact precaution but also isolation precautions along with screening for contacts [6]. Keeping in view the rapid rise of antimicrobial resistance amongst different bacteria, there is a dire need for availability of other options like daptomycin and quinupristin/dalfopristin in the country. Moreover, extra efforts are required for not only to formulate new antimicrobials but also to ensure that strict infection control precautions are exercised.

## Conclusions

Linezolid resistance amongst VRE is a rare occurrence. To our knowledge this is the first report from our region where it was isolated in a CSF shunt-related infection. Since enterococcus is spread by contact, it is very important to adhere to stringent infection control practices to avoid cross transmission between patients and healthcare workers.
